# *Dentipellicula hainanensis* (Hericiaceae, Agaricomycetes) and *Phylloporia rigida* (Hymenochaetaceae, Agaricomycetes), two new species of wood-inhabiting fungi from Hainan Province, South China

**DOI:** 10.3897/mycokeys.128.181177

**Published:** 2026-02-19

**Authors:** Chang-Ge Song, Jin-Tao Gao, Bo-Wen Yang, Ze-Feng Jia

**Affiliations:** 1 College of Life Sciences, Liaocheng University, Liaocheng 252059, China Liaocheng University Liaocheng China

**Keywords:** Macrofungi, morphology, phylogeny, taxonomy

## Abstract

Wood-rotting fungi, which are essential constituents of forest ecosystems, act as critical mediators of organic matter decomposition and material recycling within such systems. During the investigation of wood-inhabiting fungi in Hainan, China, a large number of specimens were collected. Two new species are described, based on phylogeny, morphology, host and geographic distribution. *Dentipellicula hainanensis***sp. nov**. is characterised by resupinate, soft corky and tomentose basidiomata, uneven pileal surface and ellipsoid basidiospores (2.8–)3–4.7(–5.2) × 3.2–4.2(–4.4) µm. *Phylloporia rigida***sp. nov**. is characterised by tough, fawn to reddish-brown basidiomata and ellipsoid basidiospores 2.3–4.5 × 2.2–3.8 µm. Detailed illustrations and descriptions of the novel species are provided. Phylogenetic analyses inferred from the combined ITS + nLSU datasets confirmed that the two new species are distinct within *Dentipellicula* and *Phylloporia*.

## Introduction

China harbours high diversity of wood-inhabiting fungi, with more than 1700 species documented to date (Dai 2010, 2012; Cui et al. 2019; Wu et al. 2022a, b; Liu et al. 2023; Yuan et al. 2023), the majority of which are distributed in south-western China (Wu et al. 2022b).

*Dentipellicula* Y.C. Dai & L.W. Zhou, a genus of Hericiaceae Donk, Russulales Kreisel ex P.M. Kirk, P.F. Cannon & J.C. David, is a kind of hydnoid fungi. It was established by Zhou and Dai (2013) and typified by *Dentipellicula taiwaniana* (Sheng H. Wu) Y.C. Dai & L.W. Zhou. *Dentipellicula* is characterised by hydnoid, resupinate basidiocarps with soft spines, a monomitic hyphal system with clamp connections and cyanophilous hyphae; and rough amyloid basidiospores (Ginns 1986; Zhou and Dai 2013; Chen et al. 2015; Zhou et al. 2021).

*Dentipellicula* was originally a member of *Dentipellis*. Zhou and Dai (2013) conducted a study of wood-inhabiting hydnoid species in Russulales and found that *Dentipellis taiwaniana* and *D. leptodon* (Mont.) Y.C. Dai & L.W. Zhou separated from *Dentipellis*, but phylogenetically clustered with *Wrightoporia* Pouzar. Morphologically, different from *Dentipellis* species, the generative hyphae of *D. taiwaniana* and *D. leptodon* showed a cyanophilic reaction. Meanwhile, the two species differ from *Wrightoporia* by their hydnoid hymenium and monomitic hyphal structure. Therefore, they argued that these two species did not belong to *Dentipellis* and *Wrightoporia* and established a new genus *Dentipellicula*. Subsequently, Chen et al. (2015) described *Dentipellicula austroafricana* Jia J. Chen, L.L. Shen & Y.C. Dai from South Africa, based on morphological characters and molecular data. Zhou et al. (2021) described *Dentipellicula guyanensis* Yuan Yuan, Meng Zhou, Jia J. Chen & Vlasák, based on morphological characters and ITS and nLSU sequences data from French Guiana. To date, the genus *Dentipellicula* comprises four recognised species, *D. austroafricana*, *D. guyanensis*, *D. leptodon* and *D. taiwaniana*.

The genus *Phylloporia* Murrill belongs to Hymenochaetaceae, Hymenochaetales, typified by *P. parasitica* Murrill, which was initially described from South America (Colombia) and grows on *Bignonia* sp. (Murrill 1904). *Phylloporia* species are characterised by annual or perennial, soft corky to hard corky basidiomata, tomentose to velutinate pileal surface, generative hyphae with simple septa and subglobose, ellipsoid or cylindrical, hyaline to yellowish, fairly thick-walled basidiospores. *Phylloporia* species are widely distributed in the Tropics and mostly parasitise living angiosperm trees, causing white rot (Wu et al. 2022b; Zhou et al. 2022).

With the development of molecular systematics, a series of phylogenetic studies on *Phylloporia* have emerged in recent years. Wagner and Ryvarden (2002) conducted the first monographic revision of this genus, incorporating phylogenetic data and ultimately confirmed 12 species within the genus. Olou et al. (2021) described a new species and a new record species from Benin, based on morphological characters and molecular data. Zhou et al. (2022) described two new species from the Neotropics, based on 28S ribosomal RNA phylogeny, morphology, host and geographic distribution. Subsequently, Olou et al. (2023) discovered and described a new species *Phylloporia* in Benin. Jerusalem et al. (2025) conducted a comprehensive multigene phylogeny of *Phylloporia* and described six new species from tropical Africa. To date, approximately 84 species have been recognised in *Phylloporia* worldwide, with 37 species reported from China (Wu et al. 2019a, 2020; Chamorro-Martínez et al. 2022; Zhou et al. 2022; Castro Hernández et al. 2023; Olou et al. 2023; Jerusalem et al. 2025; Shao et al. 2025).

Wood-inhabiting fungi are a morphologically, phylogenetically and ecologically diverse group, playing an integral role in wood degradation and the matter cycle within the ecological system and recognised as pivotal contributors to the intricate balance of forest ecosystems, these fungi being renowned as “key players” due to their enzymatic prowess, effectively breaking down woody components like lignin, cellulose and hemicellulose (Larsson et al. 2006; Dai et al. 2015; Dong et al. 2024, 2025; Yang et al. 2025). During our investigations of macrofungi from Hainan Province, three specimens of *Dentipellicula* and two of *Phylloporia*, exhibiting distinct morphological characteristics, were collected. The morphological observations and phylogenetic analyses, based on ITS + nLSU combined matrices were conducted to confirm the affinity of the undescribed species corresponding to *Dentipellicula* and *Phylloporia*. Accordingly, two new species are formally described and illustrated in the present study.

## Materials and methods

### Morphological studies

Specimen collection and preservation followed the methods described by Song et al. (2025). The specimens used in this study were gathered during the annual growing season of macrofungi (June to October). The specimens were dried on-site using a portable oven, to prevent putrefaction and contamination; the drying temperature was controlled at 40 °C–45 °C and the process was continued until the specimens were completely desiccated. Macromorphological descriptions were based on field notes and herbarium specimens. Detailed specimen information, such as host trees, ecological habits, geographic coordinates, location, altitude, collector and date, was recorded (Rathnayaka et al. 2025). Meanwhile, photos of the fruiting bodies and growth environment were taken by iPhone 15 Pro Max. All samples examined in this study were deposited in the Fungarium of the College of Life Sciences, Liaocheng University (LCUF). Micro-morphological data were obtained from dried specimens and observed under an Olympus BX53 compound microscope following the methods of Song et al. (2022). Basidiospores were measured from sections cut from the spines. The following abbreviations were used: IKI = Melzer’s reagent, IKI– = negative in Melzer’s reagent, KOH = 5% potassium hydroxide, CB = Cotton Blue, CB+ = cyanophilous, CB− = acyanophilous, L = mean spore length (arithmetic average of all spores), W = mean spore width (arithmetic average of all spores), Q = variation in the L/W ratios between the specimens studied and n = number of spores measured from a given number of specimens. All new fungal taxa described in this study have been formally registered in MycoBank (https://www.mycobank.org/) and the corresponding MycoBank registration numbers are provided in the taxonomic treatment section of the main text.

### Molecular study

The CTAB plant genome rapid extraction Hi-DNA-secure Plant Kit (Tiangen, Beijing, China) was used to extract total genomic DNA from dried herbarium specimens. The extracted DNA was used to perform the polymerase chain reaction (PCR) according to the manufacturer’s instructions with some modifications (Li et al. 2023; Song et al. 2025). The primer pairs, ITS5/ITS4 and LR0R/LR7, were used to amplify ITS and nLSU sequences (Song et al. 2022, 2023). The PCR process for ITS was as follows: initial denaturation at 95 °C for 3 min, followed by 35 cycles at 94 °C for 40 s, 56 °C for 45 s and 72 °C for 1 min and a final extension of 72 °C for 10 min. The PCR process for nLSU was as follows: initial denaturation at 94 °C for 1 min, followed by 35 cycles at 94 °C for 30 s, 50 °C for 1 min and 72 °C for 1.5 min and a final extension of 72 °C for 10 min. The PCR products were purified and sequenced at Beijing Genomics Institute, China, with the same primers. All newly-generated sequences were submitted to GenBank (Table 1). Other sequences in the dataset for phylogenetic analysis were downloaded from GenBank.

**Table 1.** A list of species, specimens and GenBank accession numbers of sequences used in this study.

Species	Specimen no.	Locality	GenBank accession no.		Reference
			ITS	nLSU	
*Bondarzewia montana*	DAOM F-415	Canada	DQ 200923	DQ 234539	Chen et al. (2015)
*Bondarzewia podocarpi*	Dai 9261	China	KJ 583207	KJ 583221	Chen et al. (2015)
*Dentipellicula austroafricana*	Dai 12580	South Africa	KJ 855274	KJ 855275	Chen et al. (2015)
*Dentipellicula guyanensis*	JV1808/35	Uganda	MN547359	MN547358	Zhou et al. (2021)
*Dentipellicula hainanensis*	MHN240385	China	PX612029	PX494252	This study
*Dentipellicula hainanensis*	MHN240401	China	PX612030	PX494253	This study
*Dentipellicula hainanensis*	MHN250010	China	PX612031	-	This study
*Dentipellicula leptodon*	GB 011123	China	EU 118625	EU 118625	Chen et al. (2015)
*Dentipellicula taiwaniana*	Cui 8346	China	JQ 349114	JQ 349100	Chen et al. (2015)
*Dentipellicula taiwaniana*	Dai 10867	China	JQ 349115	JQ 349101	Chen et al. (2015)
*Dentipellicula taiwaniana*	Dai 13709	China	MH 085941	MH 085957	Wang (2018)
*Dentipellicula taiwaniana*	MHN240350	China	PX612032	PX494254	This study
*Dentipellis tropicalis*	Cui 8545	China	KR 108236	KR 108240	Zhou et al. (2021)
*Dentipellis tropicalis*	He 1993	China	KR 108237	KR 108241	Zhou et al. (2021)
*Dentipellopsis dacrydicola*	Dai 12004	China	JQ 349104	JQ 349089	Zhou et al. (2021)
*Dentipellopsis dacrydicola*	Dai 12010	China	-	JQ 349090	Zhou et al. (2021)
*Fomitiporella resupinata*	DMC 476	Cameroon	KJ787822	JF712935	Zhou and Dai (2012)
*Fomitiporella sinica*	LWZ 20130809-5	China	KJ787819	KJ787810	Zhou (2014)
*Fomitiporella tenuissima*	Dai 12245	China	KC456242	KC999902	Yu et al. (2013)
*Fomitiporella umbrinella*	JV 0509/114	USA	KX181314	KX181336	Ji et al. (2017)
*Fulvifomes fastuosus*	CBS 213.36	Philippines	AY558615	AY059057	Wagner and Fischer (2002)
*Fulvifomes robiniae*	CFMR 2693	USA	KX065961	KX065995	Sayers et al. (2020)
*Fulvifomes yoroui*	OAB0097	Benin	MN017126	MN017120	Olou et al. (2019)
*Hericium coralloides*	Cui 14825	China	MH 085948	MH 085962	Wang (2018)
*Hericium coralloides*	Cui 14826	China	MH 085949	MH 085963	Wang (2018)
*Inonotus andersonii*	JV1209_66	USA	MN318443	MN318443	Olou et al. (2023)
*Inonotus hispidus*	92-829	Germany	AY624993	AF311014	Olou et al. (2023)
*Laxitextum bicolor*	NH 5166	Sweden	AF 310102	AF 310102	Zhou et al. (2021)
*Phylloporia afropectinata*	KE 16 107	Kenya	-	KY349147	Yombiyeni and Decock (2017)
*Phylloporia afropectinata*	MUCL 58359	Kenya	-	KY349148	Yombiyeni and Decock (2017)
*Phylloporia afrospathulata*	MUCL 54511	Gabon	-	KJ743248	Yombiyeni et al. (2015)
*Phylloporia afrospathulata*	MUCL 53983	Gabon	-	KJ743249	Yombiyeni et al. (2015)
*Phylloporia alyxiae*	Chen 1182	China	-	LC514407	Wu et al. (2020)
*Phylloporia alyxiae*	GC 1604-28	China	-	LC514408	Wu et al. (2020)
*Phylloporia atlantica*	JRF142	Brazil	-	MG738813	Wu et al. (2019a)
*Phylloporia atlantica*	JRF151	Brazil	-	MG738814	Wu et al. (2019a)
*Phylloporia beninensis*	OAB0142	Brazil	MW244094	MW244099	Olou et al. (2021)
*Phylloporia beninensis*	OAB0511	Benin	-	MW244096	Olou et al. (2021)
*Phylloporia bibulosa*	Ahmad27088	Pakistan	-	AF411824	Wagner and Ryvarden (2002)
*Phylloporia boldo*	CIEFAPcc532	Chile	-	MK193759	Rajchenberg et al. (2019)
*Phylloporia boldo*	CIEFAPcc584	Chile	-	MK193758	Rajchenberg et al. (2019)
*Phylloporia capucina*	Robledo1610	Argentina	-	KJ651919	Sayers et al. (2020)
*Phylloporia chrysites*	MUCL 52862	Mexico	-	HM635667	Valenzuela et al. (2011)
*Phylloporia chrysites*	MUCL 52764	Mexico	-	HM635666	Valenzuela et al. (2011)
*Phylloporia clariceae*	FLOR:51258	Brazil	-	KJ631406	Ferreira-Lopes et al. (2016)
*Phylloporia clausenae*	Yuan 3528	China	-	KJ787795	Zhou (2015a)
*Phylloporia clausenae*	Cui 8463	China	MH151186	MH165868	Zhou (2015a)
*Phylloporia crataegi*	Dai 18133	China	MH151191	MH165865	Zhou and Dai (2012)
*Phylloporia crataegi*	Dai 11016	China	-	JF712923	Zhou and Dai (2012)
*Phylloporia cryptolepidis*	GXU3569	China	OQ146995	PQ395417	Shao et al. (2025)
*Phylloporia cryptolepidis*	GXU3610	China	PQ057061	PQ063259	Shao et al. (2025)
*Phylloporia crystallina*	JV2106/102	Ecuador	-	ON006467	Zhou et al. (2022)
*Phylloporia cylindrispora*	Yuan 6144	China	-	KJ787798	Zhou (2015a)
*Phylloporia cylindrispora*	Yuan 6148	China	-	KJ787797	Zhou (2015a)
*Phylloporia cystidiolophora*	Dai 13953	China	-	MG738799	Wu et al. (2019a)
*Phylloporia cystidiolophora*	Dai 13945	China	-	MG738798	Wu et al. (2019a)
*Phylloporia dependens*	Cui 13763	China	MH151190	KX242353	Chen et al. (2017)
*Phylloporia elegans*	FLOR:51179	Brazil	-	KJ631409	Ferreira-Lopes et al. (2016)
*Phylloporia elegans*	FLOR:51178	Brazil	-	KJ631408	Ferreira-Lopes et al. (2016)
*Phylloporia ephedrae*	TAA 72-2	Turkmenistan	MH151184	AF411826	Wagner and Ryvarden (2002)
*Phylloporia flabelliforma*	MUCL 55570	Gabon	NR_154332	KU198350	Decock et al. (2015)
*Phylloporia flabelliforma*	MUCL 55569	Gabon	KU198356	KU198349	Decock et al. (2015)
*Phylloporia flacourtiae*	Yuan 6362	China	-	KJ787801	Zhou (2015a)
*Phylloporia flacourtiae*	Yuan 6360	China	-	KJ787800	Zhou (2015a)
*Phylloporia fontanesiae*	Cui 12356	China	MH151188	MH165871	Zhou and Dai (2012)
*Phylloporia fontanesiae*	Li 199	China	-	JF712925	Zhou and Dai (2012)
*Phylloporia fruticum*	MUCL 52762	Mexico	-	HM635668	Valenzuela et al. (2011)
*Phylloporia fruticum*	ENCB TR&RV858	Mexico	-	HM635669	Valenzuela et al. (2011)
*Phylloporia fulva*	MUCL 54472	Gabon	-	KJ743247	Yombiyeni et al. (2015)
*Phylloporia gabonensis*	MUCL 55572	Gabon	KU198354	KU198352	Yombiyeni et al. (2015)
*Phylloporia gabonensis*	MUCL 55571	Gabon	NR_154331	KU198353	Yombiyeni et al. (2015)
*Phylloporia gutta*	Dai 16070	China	MH151183	MH165863	Zhou and Dai (2012)
*Phylloporia gutta*	Dai 4197	China	-	JF712927	Zhou and Dai (2012)
*Phylloporia hainaniana*	Dai 9460	China	-	JF712928	Cui et al. (2010)
*Phylloporia homocarnica*	Yuan 5766	China	-	KJ787804	Zhou (2015a)
*Phylloporia homocarnica*	Yuan 5750	China	MH151195	KJ787803	Zhou (2015a)
*Phylloporia inonotoides*	MUCL 54468	China	-	KJ743250	Yombiyeni et al. (2015)
*Phylloporia lespedezae*	Dai 17065	China	MH151179	KY242602	Ren and Wu (2017)
*Phylloporia lespedezae*	Dai 17067	China	MH151180	KY242603	Ren and Wu (2017)
*Phylloporia littoralis*	MUCL: 56144	Gabon	-	KY349140	Yombiyeni and Decock (2017)
*Phylloporia littoralis*	OAB0204	Benin	MW244095	MW244098	Olou et al. (2021)
*Phylloporia lonicerae*	Dai 17900	China	MH151175	MG738802	Qin et al. (2018)
*Phylloporia lonicerae*	Dai 17899	China	MH151174	MG738801	Qin et al. (2018)
*Phylloporia manglietiae*	Cui 13709	China	MF410324	KX242358	Chen et al. (2017)
*Phylloporia memecyli*	CD-GA12-812	Gabon	-	KJ743281	Yombiyeni et al. (2015)
*Phylloporia memecyli*	MJ-GA19-091	Gabon	-	PP851484	Jerusalem et al. (2025)
*Phylloporia microspora*	LR 26485	Zimbabwe	-	PP851486	Jerusalem et al. (2025)
*Phylloporia minima*	MUCL 43132	Australia	-	PP851487	Wu et al. (2022b)
*Phylloporia minuta*	FURB 55088	Brazil	-	NG_064479	Bittencourt et al. (2018)
*Phylloporia minutipora*	Dai 16172	China	-	MH165873	Zhou (2016)
*Phylloporia minutipora*	LWZ-2016	China	-	KU904466	Sayers et al. (2020)
*Phylloporia minutipora*	Dai 9257	China	-	KU904464	Sayers et al. (2020)
*Phylloporia minutispora*	Ipulet 706	Uganda	-	JF712929	Ipulet and Ryvarden (2005)
*Phylloporia minutispora*	MUCL 52865	Congo	-	HM635671	Valenzuela et al. (2011)
*Phylloporia miomboensis*	LR 25885	Zimbabwe	-	PP851489	Jerusalem et al. (2025)
*Phylloporia montana*	BDNA2409	Brazil	-	MG738811	Wu et al. (2019b)
*Phylloporia montana*	BDNA2388	Brazil	-	MG738810	Wu et al. (2019b)
*Phylloporia mori*	Wu 1105 2	China	-	LC514412	Wu et al. (2020)
*Phylloporia mori*	Wu 1105 3	China	-	LC514413	Wu et al. (2020)
*Phylloporia moricola*	Wu 1807 1	China	-	LC589617	Wu et al. (2021)
*Phylloporia moricola*	Wu 1807 5	China	-	LC589618	Wu et al. (2021)
*Phylloporia murrayae*	Wu 1404-4	China	-	LC514409	Wu et al. (2020)
*Phylloporia murrayae*	Wu 1404-5	China	-	LC514410	Wu et al. (2020)
*Phylloporia mutabilis*	OAB0643	Benin	OR096158	OR096136	Olou et al. (2023)
*Phylloporia mutabilis*	OAB0666	Benin	OR096159	OR096137	Olou et al. (2023)
*Phylloporia nandinae*	Dai 10588	China	-	JF712930	Zhou and Dai (2012)
*Phylloporia nandinae*	Dai 10625	China	-	JF712931	Zhou and Dai (2012)
*Phylloporia nodostipitata*	FLOR:51173	Brazil	KJ639057	KJ631412	Ferreira-Lopes et al. (2016)
*Phylloporia nodostipitata*	FLOR:51175	Brazil	-	KJ631413	Ferreira-Lopes et al. (2016)
*Phylloporia nouraguensis*	MUCL/FG-11-404	Guyana	-	KC136223	Decock et al. (2013)
*Phylloporia nouraguensis*	MUCL/FG-11-409	Guyana	-	KC136224	Decock et al. (2013)
*Phylloporia oblongospora*	Zhou 179	China	MH151197	JF712932	Cui et al. (2010)
*Phylloporia oreophila*	Cui 2219	China	MH151196	JF712933	Zhou and Dai (2012)
*Phylloporia oreophila*	Cui 9503	China	-	JF712934	Zhou and Dai (2012)
*Phylloporia oropheae*	GXU3728	China	OQ146998	OQ147002	Shao et al. (2025)
*Phylloporia oropheae*	GXU4511	China	OQ146997	PQ395421	Shao et al. (2025)
*Phylloporia osmanthi*	Yuan 5655	China	-	KF729938	Zhou (2015b)
*Phylloporia parasitica*	LR 19843	Argentina	KU198361	-	Decock et al. (2015)
*Phylloporia pectinata*	R.Coveny 113	Australia	-	AF411823	Wagner and Ryvarden (2002)
*Phylloporia pendula*	Cui 13691	China	-	KX242357	Chen et al. (2017)
*Phylloporia pendula*	Cui 13876	China	-	KX901670	Chen et al. (2017)
*Phylloporia perangusta*	Dai 18139	China	MH151169	MG738803	Wu et al. (2019a)
*Phylloporia pseudopectinata*	Cui 13746	China	-	KX242355	Chen et al. (2017)
*Phylloporia pseudopectinata*	Cui 13749	China	-	KX242356	Chen et al. (2017)
*Phylloporia pseudoweberiana*	KE15-02	Kenya	-	KU358722	Jerusalem et al. (2025)
*Phylloporia pulla*	Cui 5251	China	-	KU904468	Zhou (2016)
*Phylloporia pulla*	Dai 9627	China	-	KU904469	Zhou (2016)
*Phylloporia radiata*	LWZ-2016a	China	-	KU904470	Zhou (2016)
*Phylloporia rattanicola*	Dai 18233	China	-	MG738807	Wu et al. (2019a)
*Phylloporia rattanicola*	Dai 18235	China	MH151172	MG738808	Wu et al. (2019a)
*Phylloporia ribis*	82-828	Germany	-	AF311040	Wagner and Ryvarden (2002)
*Phylloporia rigida*	MHN240359	China	PX612033	-	This study
*Phylloporia rigida*	MHN240360	China	PX612034	-	This study
*Phylloporia rinoreae*	MUCL: 56283	Gabon	-	MN243144	Jerusalem et al. (2019)
*Phylloporia rinoreae*	MUCL: 57328	Gabon	-	MN243146	Jerusalem et al. (2019)
*Phylloporia rubiacearum*	Chen 3583	China	-	LC514416	Wu et al. (2020)
*Phylloporia rubiacearum*	Chen 3584	China	-	LC514417	Wu et al. (2020)
*Phylloporia rzedowskii*	MUCL 52859	Mexico	-	HM635673	Valenzuela et al. (2011)
*Phylloporia rzedowskii*	MUCL 52860	Mexico	-	HM635674	Valenzuela et al. (2011)
*Phylloporia sumacoensis*	JV2109/73	Ecuador	-	ON006468	Zhou et al. (2022)
*Phylloporia solicola*	JRF145	Brazil	-	MG738815	Wu et al. (2019a)
*Phylloporia spathulata*	Chay 456	Mexico	-	AF411822	Ferreira-Lopes et al. (2016)
*Phylloporia splendida*	Dai 6282	China	-	MG738805	Wu et al. (2019a)
*Phylloporia splendida*	Cui 8429	China	-	MG738804	Wu et al. (2019a)
*Phylloporia tabernaemontanae*	Dai 18852	China	-	MZ437409	Wu et al. (2022b)
*Phylloporia tabernaemontanae*	Dai 18853	China	-	MZ437410	Wu et al. (2022b)
*Phylloporia terrestris*	Yuan 5738	China	-	KC778784	Zhou (2015b)
*Phylloporia terrestris*	He 2359	China	MH151189	MH165869	Zhou (2015b)
*Phylloporia tiliae*	Yuan 5491	China	-	KJ787805	Zhou (2013)
*Phylloporia ulloai*	MUCL 52866	Mexico	-	HM635677	Valenzuela et al. (2011)
*Phylloporia ulloai*	MUCL 52867	Mexico	-	HM635678	Valenzuela et al. (2011)
*Phylloporia verae-crucis*	F19-159	Mexico	-	PP851647	Jerusalem et al. (2025)
*Phylloporia warneckeicola*	GA12-813	Gabon	-	KJ743253	Jerusalem et al. (2025)
*Phylloporia warneckeicola*	GA12-814	Gabon	-	KJ743256	Jerusalem et al. (2025)
*Phylloporia weberiana*	Dai 9242	China	-	JF712936	Sayers et al. (2020)
*Phylloporia yuchengii*	YG 051	Uzbekistan	-	KM264325	Gafforov et al. (2014)
*Pseudowrightoporia crassihypha*	Yuan 6247	China	KM 107873	KM 107892	Wang (2018)
*Pseudowrightoporia crassihypha*	Yuan 5884	China	KM 107872	KM 107891	Wang (2018)
*Wrightoporiopsis biennis*	Cui 8457	China	KJ 807066	KJ 807074	Zhou et al. (2021)
*Wrightoporiopsis biennis*	Cui 8506	China	KJ 807067	KJ 807075	Wang (2018)

New species are shown in bold.

### Phylogenetic analyses

The phylogenetic relationships of *Dentipellicula* and *Phylloporia* were inferred, based on the combined ITS + nLSU sequence datasets. The datasets were aligned in MAFFT 7 (Katoh and Standley 2013) and manually adjusted in BioEdit (Hall 1999). The sequences of *Bondarzewia podocarpi* Y.C. Dai & B.K. Cui and *B. occidentalis* Jia J. Chen, B.K. Cui & Y.C. Dai were used as the outgroups for *Dentipellicula*, according to Zhou and Dai (2013). The sequences of *Inonotus andersonii* (Ellis & Everh.) Nikol. and *I. hispidus* (Bull.) P. Karst. were used as the outgroups for *Phylloporia*, according to Olou et al. (2023). Alignments were concatenated in Mesquite v. 3.2. The best-fit model of nucleotide evolution for the datasets was selected with AIC (Akaike Information Criterion) using MrModelTest 2.3 (Posada and Crandall 1998).

Maximum Likelihood (ML) analysis was performed in RAxML v.7.2.8 with a GTR + G + I model (Stamatakis 2006). All model parameters were estimated by the programme, but only the best Maximum-Likelihood tree from all searches was kept. MrModelTest 2.3 (Posada and Crandall 1998; Nylander 2004) was used to determine the best-fit evolution model for each dataset for Bayesian Inference (BI).

BI was performed using MrBayes 3.2.6 with two independent runs, each one beginning from random trees with four simultaneous independent chains, performing 5 million generations, sampling every 100 generations (Ronquist and Huelsenbeck 2003). The first 25% of the sampled trees were discarded as burn-in and a majority rule consensus tree of all remaining trees was calculated.

Branches that received bootstrap support for Maximum Likelihood (ML) and Bayesian posterior probabilities (BPP) greater than or equal to 50% (ML) and 0.95 (BPP) were regarded as prominently supported. Phylogenetic trees were visualised using FigTree v.1.4.2 (http://tree.bio.ed.ac.uk/software/figtree/, accessed on 18 November 2025).

## Results

### BLAST search results

BLAST searches of the ITS sequences for all specimens (Table 2) showed that MHN240385, MHN240401 and MHN250010 matched *Wrightoporiopsis amylohypha* with query cover of 95%, 95% and 85% and percentage identity of 83.31%, 83.31% and 90.68%, respectively. Specimens MHN240359 and MHN240360 had the top hits to *Phylloporia* sp., with 100%/99% query cover and 87.71%/87.08% percentage identity. These results provided preliminary molecular evidence for the taxonomic assignment of the specimens, supporting subsequent phylogenetic analysis.

**Table 2.** Results of BLAST search for each specimen.

Specimen no.	BLAST results (based on ITS)	Query Cover	Per. Ident
MHN240385	*Wrightoporiopsis amylohypha*	95%	83.31%
MHN240401	*Wrightoporiopsis amylohypha*	95%	83.31%
MHN250010	*Wrightoporiopsis amylohypha*	85%	90.68%
MHN240359	*Phylloporia* sp.	100%	87.71%
MHN240360	*Phylloporia* sp.	99%	87.08%

### Phylogenetic analyses

**Figure 1.**
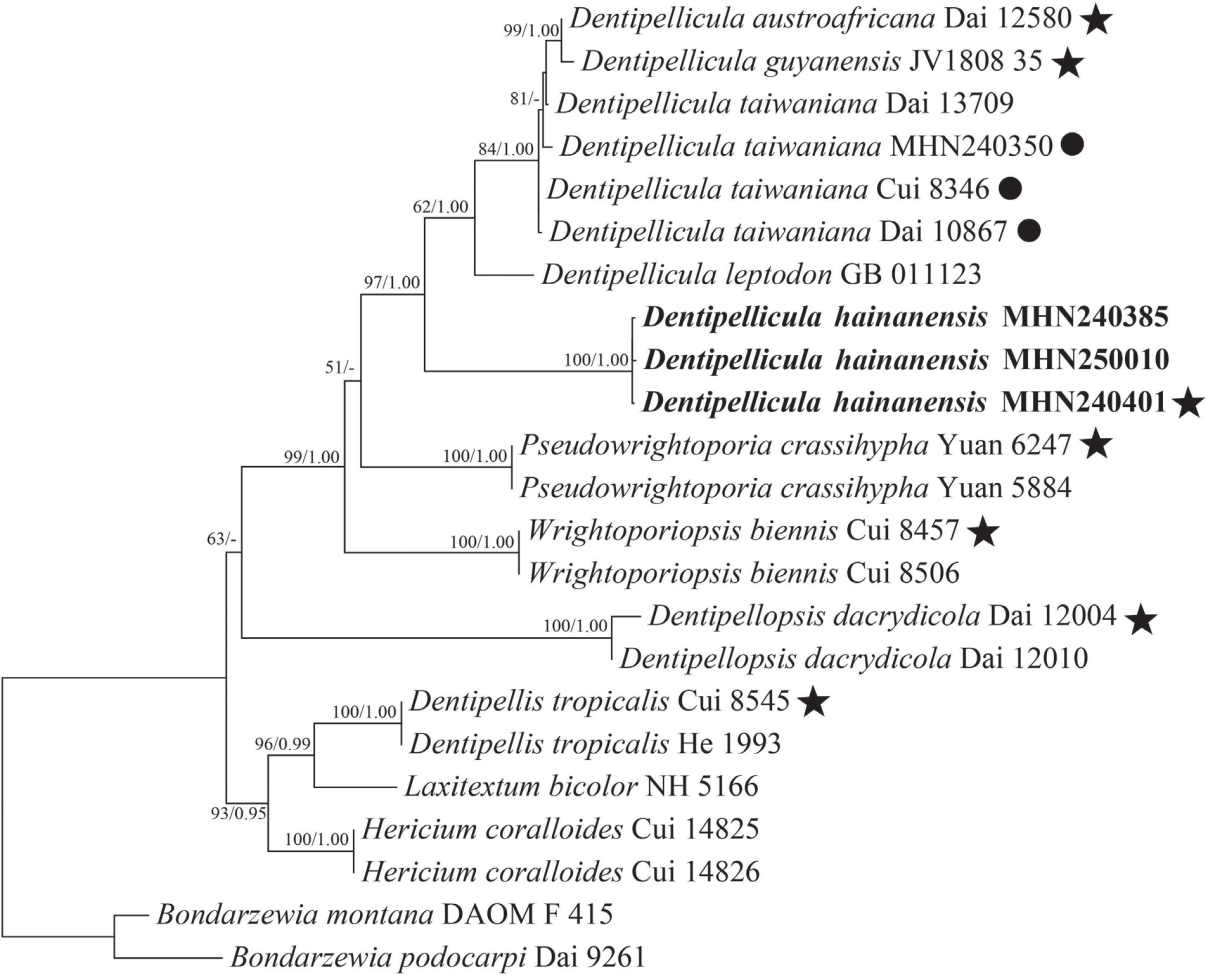
Maximum Likelihood tree illustrating the phylogeny of *Dentipellicula*, based on the combined sequence dataset of ITS + nLSU. Branches are labelled with Maximum Likelihood bootstrap higher than 50% and Bayesian posterior probabilities more than 0.95, respectively. Bold names = new species. Black stars: represent type sequences. Filled circles: represent type species.

The combined ITS + nLSU dataset of Hericiaceae included sequences from 23 fungal strains representing 13 taxa. The best-fit model for each partition was TN+F+G4 for ITS and TN+F+I+G4 for nLSU. Bayesian analysis yielded a topology similar to that from the ML analysis. The Bayesian analysis yielded a concordant topology with an average standard deviation of split frequencies of 0.009999. Only the ML tree is provided in Fig. 1 and the ML (50%) and BI (0.95) are shown at the nodes.

**Figure 2.**
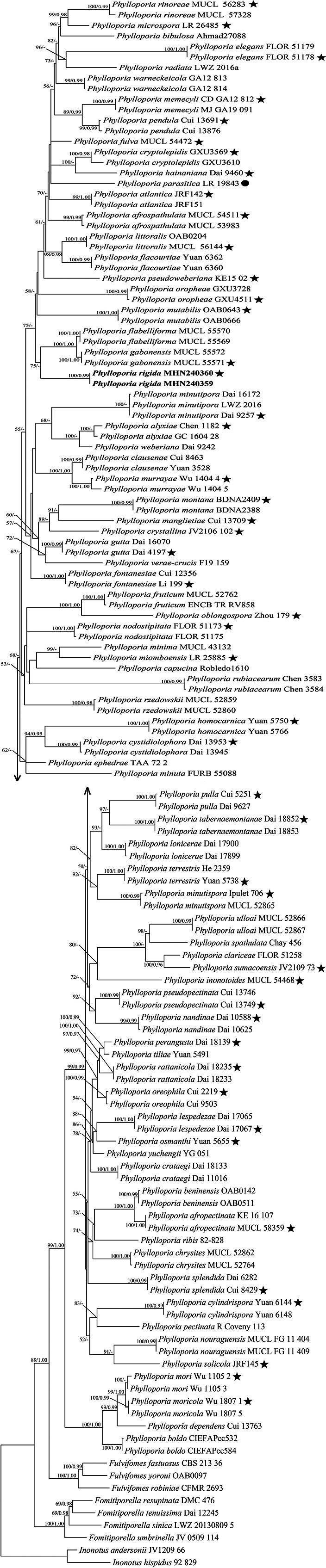
Maximum Likelihood tree illustrating the phylogeny of *Phylloporia*, based on the combined sequence dataset of ITS+nLSU. Branches are labelled with Maximum Likelihood bootstrap higher than 50% and Bayesian posterior probabilities more than 0.95, respectively. Bold names = new species. Black stars: represent type sequences. Filled circles: represent type species.

The combined ITS + nLSU dataset of *Phylloporia* included sequences from 131 fungal strains representing 85 taxa. The best models for each region of the combined ITS + nLSU sequence dataset, as estimated and applied in the Bayesian analysis, were both GTR + I + G models. Bayesian analysis yielded a topology similar to that from the ML analysis. The Bayesian analysis resulted in a concordant topology with an average standard deviation of split frequencies = 0.009999. Only the ML tree is provided in Fig. 2 and the ML (50%) and BI (0.95) are shown at the nodes.

Phylogenetic analysis, based on the combined ITS + nLSU dataset (Fig. 1), provided more robust and accurate taxonomic placement of the specimens compared to preliminary BLAST searches. While BLAST results initially matched specimens MHN240385, MHN240401 and MHN250010 to *Wrightoporiopsis*, the phylogenetic tree clearly resolved the three specimens as a distinct, well-supported lineage (100%ML/1.00BI) representing the new species *Dentipellicula hainanensis*, which clustered within *Dentipellicula* rather than *Wrightoporiopsis*.

For specimens MHN240359 and MHN240360, BLAST searches targeting their ITS sequences initially identified *Phylloporia* sp. as the top homologous matches. This initial taxonomic assignment was robustly supported by our phylogenetic analysis of the combined ITS + nLSU dataset (Fig. 2), which confidently positioned both specimens within *Phylloporia*. Notably, the two specimens clustered together in a well-supported, monophyletic clade (100% ML/0.99 BI), indicating that they merit recognition as a new species in *Phylloporia*.

### Taxonomy

#### Dentipellicula hainanensis

Taxon classificationFungiRussulalesHericiaceae

C.G. Song & Z.F. Jia sp. nov.

E954190B-1775-597C-857C-9F2A85F5EA77

MycoBank No: 861880

Figs 3, 4

##### Diagnosis.

Differs from other *Dentipellicula* species by its resupinate, soft corky and tomentose, basidiomata, uneven pileal surface and the presence of clamp connections in generative hyphae.

**Figure 3.**
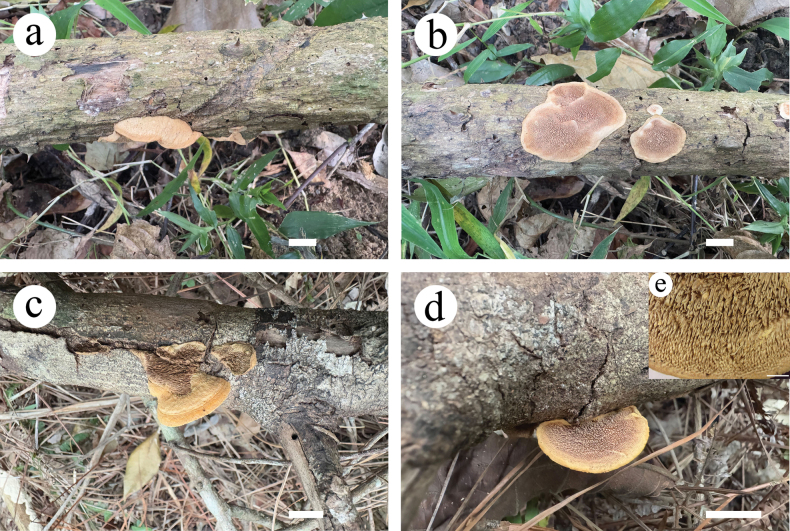
Basidiocarps of *Dentipellicula hainanensis*. Scale bars: 2 cm (**a–d**); 0.3 cm (**e**).

**Figure 4.**
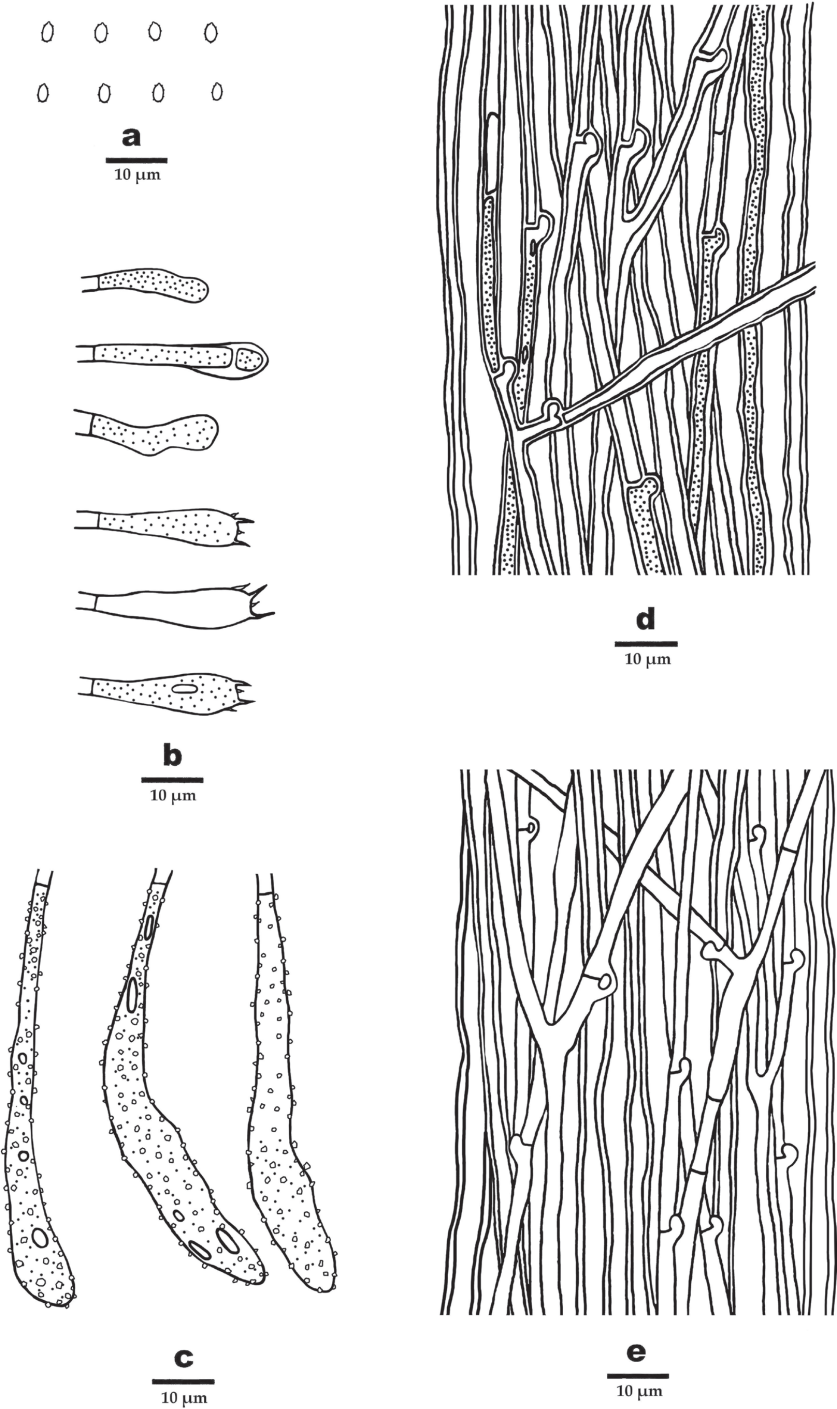
Microscopic structures of *Dentipellicula hainanensis* (drawn from the holotype). **a**. Basidiospores; **b**. Basidia and basidioles; **c**. Gloeocystidia; **d**. Hyphae from context; **e**. Hyphae from spines.

##### Type.

China • Hainan Province, Yinggeling National Nature Reserve, on fallen wood, alt. 670 m, 19°02'24"N, 109°34'12"E, 31 December 2024, MHN240401.

##### Etymology.

*Hainanensis* (Lat.) refers to the holotype locality of the species in Hainan Province.

##### Fruiting body.

Basidiomata annual, resupinate, without odour or taste. Pileus fan-shaped to nearly circular, up to 2.5 cm in diam., 0.3 cm thick at centre. Pileal surface greyish-orange (5B5) to light orange (6A5) to pinkish-buff, uneven, azonate, tomentose; margin darker than the middle, greyish-orange (6B5) buff, up to 0.35 cm wide. Fresh spines soft, light orange (6A5) to greyish-orange (6B5) when fresh, becoming fragile, upon drying, up to 2 mm long, 7–9 per mm across base. Subiculum soft corky, concolorous with pileal surface, up to 0.2 cm thick.

##### Microstructure.

Hyphal system monomitic; generative hyphae with clamp connections; IKI–, CB–; tissue unchanged in KOH. Generative hyphae in context hyaline, thick-walled, occasionally branched, interwoven to regularly arranged and 3–6 µm in diam. Generative hyphae in spines hyaline, thin- to thick-walled, occasionally branched, regularly arranged and 3–5 µm in diam. Gloeocystidia present, rooting deep from the trama, often encrusted, 8–11.6 µm in diam., cystidioles absent. Basidia clavate, 4-spored, 16–23 × 3.8–7.5 µm; basidioles similar to basidia in shape. Basidiospores, hyaline, ellipsoid, thick-walled, echinulate, IKI+, CB–, (2.8–)3–4.7(–5.2) × 3.2–4.2(–4.4) µm, L = 4 µm, W = 3.6 µm, Q = 1.03–1.32 (n = 90/3).

##### Additional specimens (paratypes) examined.

• Hainan Province, Changjiang County, Bawangling, Yajia Scenic Area, on a branch, alt. 630 m, 19°05'06"N, 109°07'22.8"E, 30 December 2024, MHN240385; Yinggeling National Nature Reserve, on fallen wood, alt. 670 m, 19°03'10.8"N, 109°33'50.4"E, 1 January 2025, MHN250010.

#### Phylloporia rigida

Taxon classificationFungiHymenochaetalesHymenochaetaceae

C.G. Song & Z.F. Jia sp. nov.

6478CB6D-50D3-53DA-9B32-7E5EB664CCF5

MycoBank No: 861881

Figs 5, 6

##### Diagnosis.

Differs from other *Phylloporia* species by its pileate, sessile basidiomata, fawn to reddish-brown and fan-shaped pileal surface, salmon to peach pileal margin, cream to buff yellow tubes and monomitic hyphal system and ellipsoid, thick-walled basidiospores.

**Figure 5.**
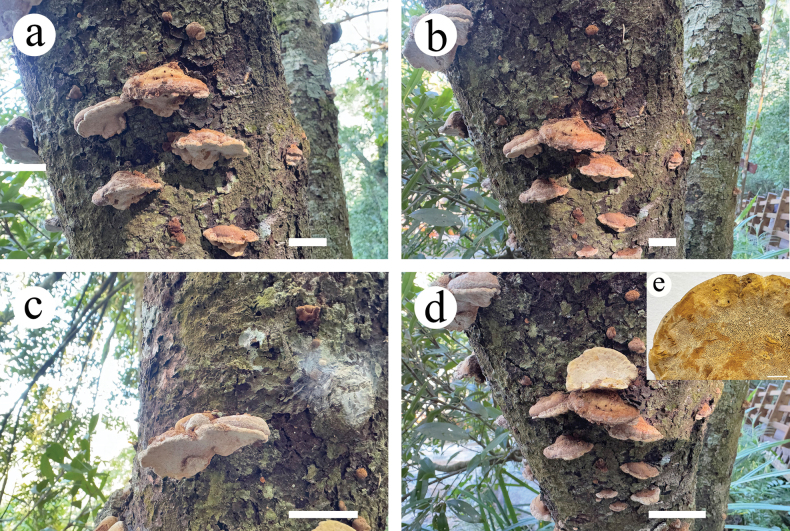
Basidiocarps of *Phylloporia rigida*. Scale bars: 2 cm (**a–d**); 0.3 cm (**e**).

**Figure 6.**
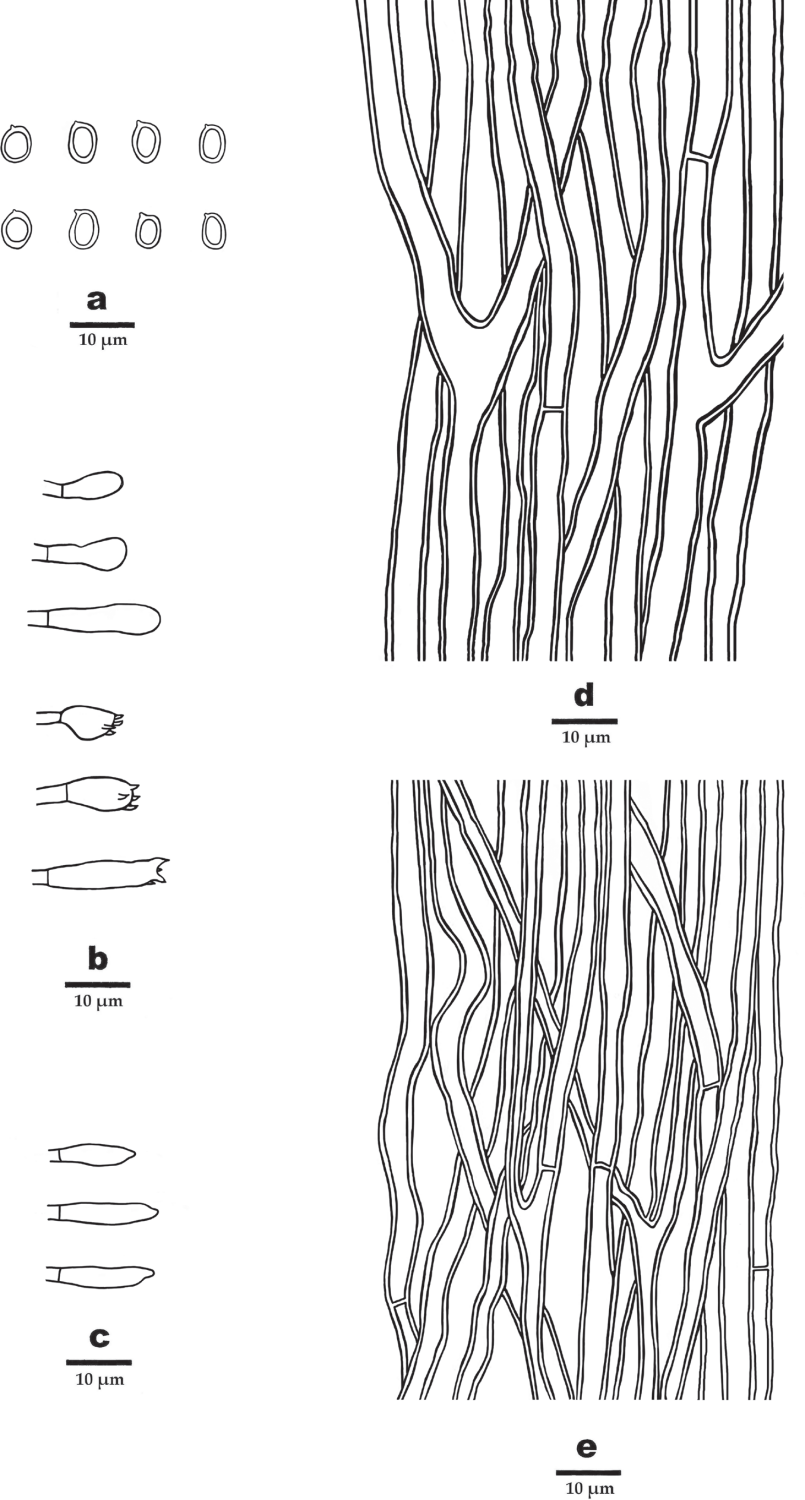
Microscopic structures of *Phylloporia rigida* (drawn from the holotype). **a**. Basidiospores; **b**. Basidia and basidioles; **c**. Cystidioles; **d**. Hyphae from context; **e**. Hyphae from spines.

##### Type.

China • Hainan Province, Changjiang County, Bawangling Yajia Scenic Area, on the living tree of *Garcinia oblongifolia* Champ. ex Benth., alt. 495 m, 19°05'06"N, 109°07'22.8"E, 30 December 2024, MHN240360.

##### Etymology.

*Rigida* (Lat.) refers to the rigid basidiomata.

##### Fruiting body.

Basidiomata annual, epixylous, pileate, sessile, without odour or taste. Pileus applanate to nearly half round, up to 4 cm in diam., 2 cm thick at centre. Pileal surface greyish-orange (6B5), uneven, azonate, fibrous to tomentose; margin greyish-orange (6B4), greyish-red (7B6) to dull red (8B3), wave, up to 1.2 cm wide. Tube light orange (6A4) when fresh, tough upon drying, up to 1 mm long, 5–7 per mm. Context, rigid, duplex, with a lower corky context separated from an upper trichoderm by a thin black line, up to 0.4 cm thick.

##### Microstructure.

Hyphal system monomitic; generative hyphae with simple septa; IKI–, CB–; tissue darker in KOH. Generative hyphae in context yellowish-brown, slightly thick-walled, occasionally branched, interwoven to regularly arranged and 3–8 µm in diam. Generative hyphae in tubes yellowish-brown, thick-walled, often branched, regularly arranged and 2–6 µm in diam. Gloeocystidia absent. Cystidioles present, 10.5–13.5 × 1–3.5 µm in diam. Basidia clavate, 4-spored, 9–13 × 2.5–5 µm; basidioles similar to basidia in shape. Basidiospores ellipsoid, oblong-ellipsoid to cylindrical, yellow-brown, ellipsoid, thick-walled, IKI–, CB–, 2.3–4.5 × 2.2–3.8 µm, L = 3.31 µm, W = 2.83 µm, Q = 1–1.32 (n = 60/2).

##### Additional specimen (paratype) examined.

China • Hainan Province, Changjiang County, Bawangling Yajia Scenic Area, on a living tree of *Garcinia oblongifolia*, alt. 495 m, 19°05'06"N, 109°07'22.8"E, 30 December 2024, MHN240359.

## Discussion

In the present study, phylogenetic analysis of the combined ITS and nLSU sequences revealed that *Dentipellicula hainanensis* is distantly related to *Dentipellis*, *Hericium* and *Laxitextum*, but clusters within the *Dentipellicula* clade (Fig. 1).

*Dentipellicula hainanensis* formed a single lineage, different from other species of *Dentipellicula* in our phylogenetic analyses (Fig. 1). Morphologically, *D. hainanensis* might be confused with *D. leptodon* in having cottony pileus, pale yellow spines and the presence of clamp connections in generative hyphae (Ginns 1986). However, *D. leptodon* is distinguished by its white pileus margin, longer spines measuring 3–5 mm, longer basidia measuring 20–26 × 4–5 µm and narrower basidiospores measuring 3.7 × 2.8 µm (Ginns 1986). The prevalence of newly-described species in subtropical and tropical areas indicates a remarkable richness of *Phylloporia* in these regions (Anonymous 1997; Decock et al. 2015; Olou et al. 2021, 2023; Wu et al. 2022b; Zhou et al. 2022; Jerusalem et al. 2025). In the present study, one new species of *Phylloporia* is described from Hainan Province, based on morphological characters and phylogenetic analyses of the combined ITS + nLSU sequences. The main morphological characteristics of each *Phylloporia* species from China were summarised in Table 3.

**Table 3.** The ecological habits and main morphological characters of species in *Phylloporia* from China.

Species	Ecological habits	Basidiomata	Pores	Context	Hyphal system	Cystidioles	Basidiospores (µm)	Wu et al. (2020)
*P. alyxiae*	on living *Alyxia insularis*	perennial, pileate	6–8 per mm	duplex with a black line	dimitic	present	(2.8−)3–3.2(−3.5) × (2−)2.2–2.7	Zhou (2015a)
*P. clausenae*	on living *Crataegus*	annual, pileate	8–9 per mm	duplex with two black zones	monomitic	absent	3–3.5 × 2–3	Zhou and Dai (2012)
*P. crataegi*	on living *Crataegus*	perennial, pileate	7–9 per mm	duplex with a black line	monomitic	absent	2.5–3.3 × 2–2.8	Shao et al. (2025)
*P. cryptolepidis*	on the branch of living *Cryptolepis buchananii*	annual, pileate	5–6 per mm	duplex with a black line	monomitic	absent	(3.2–)3.6–4.6(–4.7) × (2.2 )2.5–3.5(–3.9)	Zhou (2015a)
*P. cylindrispora*	on living angiosperm	annual, pileate	6–8 per mm	duplex with a black line	monomitic	absent	3.5–4 × 1.5–2	Wu et al. (2019b)
*P. cystidiolophora*	on living angiosperm	annual, pileate	8–10 per mm	duplex with a black line	monomitic	present	2.5–3 × 2.1–2.8	Liu et al. (2015)
*P. dependens*	on living angiosperm	perennial, pileate to pendent	7–9 per mm	homogenous	monomitic	present	3–3.4 × 2.7–3	Zhou (2015a)
*P. flacourtiae*	on living *Flacourtia*	annual, pileate	5–7 per mm	duplex without black line	monomitic	absent	3.7–4.4 × 2.5–3.2	Zhou and Dai (2012)
*P. fontanesiae*	on living *Fontanesia*	annual, pileate	10–12 per mm	duplex with a black line	monomitic	absent	2.5–3 × 2–2.5	Zhou and Dai (2012)
*P. gutta*	on dead *Abelia* and other angiosperm	perennial, pileate	7–9 per mm	duplex with a black line	monomitic	absent	3–4 × 2–2.5	Cui et al. (2010)
*P. hainaniana*	on living angiosperm	annual, pileate	4–6 per mm	duplex with a black line	monomitic	absent	4.6–5.6 × 3–3.6	Zhou (2015a)
*P. homocarnica*	on dead angiosperm	annual, pileate	4–6 per mm	homogenous	monomitic	absent	4–4.9 × 2.5–3	Ren and Wu (2017)
*P. lespedezae*	on living *Lespedeza*	annual, pileate	8–9 per mm	duplex with a black line	monomitic	present	3.1–3.7 × 2.2–2.7	Qin et al. (2018)
*P. lonicerae*	on living *Lonicera*	perennial, pileate	6–8 per mm	duplex with a black line	dimitic	absent	3–3.3 × 2.1–2.5	Chen et al. (2017)
*P. manglietiae*	on living *Manglietia*	perennial, pileate	6–8 per mm	duplex with a black line	monomitic	absent	3–3.5 × 2–2.5	Zhou (2016)
*P. minutipora*	on living angiosperm	annual, pileate	12–15 per mm	duplex with a black line	dimitic	absent	2.5–3 × 2–2.4	Wu et al. (2022b)
*P. minutissima*	on living angiosperm shrub	annual, pileate	12–13 per mm	duplex with a black line	monomitic	absent	(2.9−)3 3.5(−3.6) × (1.8−)2–2.3(−2.5)	Wu et al. (2020)
*P. mori*	on fallen trunk of *Morus*	perennial, resupinate to effused reflexed	7–9 per mm	homogeneous	dimitic	present	(3.8−)4–4.7(−5) × 3.1–3.6(−4)	Wu et al. (2021)
*P. moricola*	on living tree of *Morus*	perennial, resupinate to effused reflexed	7–9 per mm	duplex with a black line	dimitic	absent	(3.2–)3.5–4 × (2.5 )2.8–3.1(−3.3)	Wu et al. (2020)
*P. murrayae*	on living *Murraya paniculata*	perennial, pileate	8–10 per mm	duplex without black line	dimitic	absent	(3−)3.2–3.7 × (2.5−)2.8–3(−3.2)	Zhou and Dai (2012)
*P. nandinae*	on living *Nandina*	annual, pileate	5–6 per mm	duplex with a black line	monomitic	absent	3.5–4.2 × 2–2.5	Cui et al. (2010)
*P. oblongospora*	on living angiosperm	annual, pileate	2–4 per mm	homogenous	monomitic	absent	4–4.8 × 2–2.5	Zhou and Dai (2012)
*P. oreophila*	on living angiosperm	annual, pileate	7–9 per mm	duplex with a black line	monomitic	absent	3–3.7 × 2–3	Shao et al. (2025)
*P. oropheae*	on branch of living *orophea hainanensis*	annual, pileate	3–4 per mm	duplex with a black line	dimitic	absent	(4.1–) 4.2–5.3(–5.7) × (2.5–)2.6–3.8(–3.9)	Zhou (2015b)
*P. osmanthi*	on living *Osmanthus*	annual, pileate	7–9 per mm	duplex with a black line	monomitic	present	2.9–3.4 × 2–2.6	Wu et al. (2019b)
*P. perangusta*	on living angiosperm	perennial, pileate	9–11 per mm	duplex with a black line	dimitic	present	2.5–2.9 × 1.8–2	Chen et al. (2017)
*P. pendula*	on living angiosperm	annual, pileate	7–9 per mm	duplex with a black line	dimitic	absent	3.5–4 × 2.5–3	Chen et al. (2017)
*P. pseudopectinata*	on living angiosperm	annual, pileate	8–9 per mm	duplex with a black line	dimitic	absent	3–3.5 × 2–3	Yombiyeni et al. (2015)
*P. pulla*	unknown substrate	Annual, pileate	11–12 per mm	duplex	dimitic	absent	2.8–3.3 × 2.3–2.8	Zhou (2016)
*P. radiata*	on living lianas	annual, pileate	8–10 per mm	duplex with a black line	monomitic	absent	2.5–3.5 × 2–2.5	Wu et al. (2019b)
*P. rattanicola*	on lianas	perennial, pileate	9–11 per mm	duplex with a black line	dimitic	present	2.9–3.1 × 2–2.1	This study
*P. rigida*	on living *Garcinia oblongifolia*	annual, pileate	5–7 per mm	duplex with a black line	monomitic	present	2.3–4.5 × 2.2–3.8	Wu et al. (2020)
*P. rubiacearum*	on living trees of Rubiaceae	perennial, pileate	7–9 per mm	duplex with a black line	dimitic	present	3–3.5 × (2.2−)2.3–2.8(−2.9)	Wu et al. (2019b)
*P. splendida*	on living angiosperm	annual, pileate	9–10 per mm	duplex with a black line	monomitic	present	3.2–4 × 2.1–2.8	Wu et al. (2019b)
*P. subpulla*	on living angiosperm	perennial, pileate	13–16 per mm	duplex with a black line	dimitic	absent	2.7–3 × 1.9–2.1	Zhou (2015b)
*P. terrestris*	on ground	annual, stipitate	10–14 per mm	duplex with a black line	monomitic	absent	2.5–3.3 × 1.8–2.5	Zhou (2013)
*P. tiliae*	on living *Tilia*	perennial, pileate	9–12 per mm	duplex with a black line	monomitic	absent	3–3.4 × 2–2.5	Dai (2010)
*P. weberiana*	on living angiosperm	annual, pileate	6–8 per mm	duplex with a black line	monomitic	absent	3.4 4.1 × 2.2–3	Dai (2010)

*Phylloporia rigida* grouped with *Phylloporia flabelliformis* Decock & Yombiy. and *Phylloporia gabonensis* Decock & Yombiy. in our phylogenetic analyses (Fig. 2). Morphologically, *P. flabelliformis* and *P. gabonensis* are similar to *P. rigida* in having pileate, sessile basidiomata and cork-coloured pileal surface and similar basidia. However, *P. flabelliformis* is distinguished by its shiny, smooth pileal surface, thin context, white pileal margin and smaller basidiospores, measuring (3–)3.3–4 × (2.2–)2.5–2.8(–3) µm (Decock et al. 2015). *Phylloporia gabonensis* is distinguished by its dull, smooth pileal surface, thin, irregular, pale yellow to pale greyish-orange pileal margin and slightly smaller basidiospores, measuring 3.7–4.2(–5) × 2.7–3(–3.3) µm (Decock et al. 2015).

For ecological traits, *Dentipellicula* species predominantly grow on angiosperms and are frequently found on fallen trunks and dead trees (Chen et al. 2015; Wang 2018; Zhou et al. 2021). However, the genus *Phylloporia* displays marginally distinct ecological traits. Most *Phylloporia* species are parasitic on living hardwood hosts; however, a few species grow on deadwood and speciation in this genus is likely promoted by the colonisation of new hosts and the adaptive evolution that follows (Wu et al. 2019a). High levels of host specificity are displayed by the majority of Phylloporia species that parasitise living hardwood hosts (Table 2) (Wagner and Ryvarden 2002). It indicated that species in *Phylloporia* are host-biased, which can be used as an auxiliary basis for species discovery and identification.

### Key to accepted species of *Dentipellicula*

**Table:** 

1	Pileal surface buff yellow to pinkish-buff	***D. hainanensis***
–	Pileal surface with different colour	**2**
2	Basidiospores > 3.5 µm long	***D. leptodon***
–	Basidiospores < 3.5 µm long	**3**
3	Spines 8–10 per mm	***D. austroafricana***
–	Spines 5–7 per mm	**4**
4	Basidiocarps resupinate; gloeoplerous hyphae present	***D. guyanensis***
–	Basidiocarps effused-reflexed; gloeoplerous hyphae absent	***D. taiwaniana***

### Key to species of *Phylloporia* from China

**Table:** 

1	Basidiomata stipitate	***P. terrestris***
–	Basidiomata sessile	**2**
2	Hyphal system dimitic	**3**
–	Hyphal system monomitic	**16**
3	Pores > 12 per mm	**4**
–	Pores < 12 per mm	**5**
4	Basidiomata perennial; basidiospores 1.9–2.1 μm wide, basidia 11–13 μm long	***P. subpulla***
–	Basidiomata annual; basidiospores 2–2.5 μm wide, basidia 5–7 μm long	***P. minutipora***
5	Basidiomata perennial	**6**
–	Basidiomata annual	**13**
6	Cystidioles absent	**7**
–	Cystidioles present	**9**
7	Basidiocarps resupinate to effused reflexed	***P. moricola***
–	Basidiocarps pileate	**8**
8	Basidiomata pendent, basidia 7–18 μm long	***P. murrayae***
–	Basidiomata not pendent, basidia 13–15 μm long	***P. Lonicerae***
9	Basidiocarps resupinate to effused reflexed	***P. mori***
–	Basidiocarps pileate	**10**
10	Pores > 9 per mm	**11**
–	Pores < 9 per mm	**12**
11	Upper tomentum up to 2 mm thick; basidiospores 2.5–2.9 μm long	***P. perangusta***
–	Upper trichoderm up to 0.5 mm thick; basidiospores 2.9–3.1 μm long	***P. rattanicola***
12	Pores 6–8 per mm	***P. alyxiae***
–	Pores 7–9 per mm	***P. rubiacearum***
13	Pores > 9 per mm	***P. pulla***
–	Pores < 9 per mm	**14**
14	Pores > 4 per mm	**15**
–	Pores < 4 per mm	***P. oropheae***
15	Basidiomata pendent; basidiospores 3.5–4 μm long	***P. pendula***
–	Basidiomata not pendent; basidiospores 2.8–3.5 μm long	***P. pseudopectinata***
16	Context homogeneous	**17**
–	Context duplex	**19**
17	Basidiospores broadly ellipsoid to subglobose	***P. dependens***
–	Basidiospores ellipsoid to oblong-ellipsoid	**18**
18	Pores 4–6 per mm; basidiospores 2.5–3 μm wide	***P. homocarnica***
–	Pores 2–4 per mm; basidiospores 2–2.5 μm wide	***P. oblongospora***
19	Black line absent in context	**20**
–	Black line present in context	**21**
20	Basidiospores 2–2.5 μm wide	***P. nandinae***
–	Basidiospores 2.5–3.5 μm wide	***P. flacourtiae***
21	Basidiospores broadly ellipsoid to subglobose	**22**
–	Basidiospores ellipsoid, oblong-ellipsoid to cylindrical	**29**
22	Cystidioles present	**23**
–	Cystidioles absent	**24**
23	Basidiospores broadly ellipsoid, 3.1–3.7 μm long	***P. lespedezae***
–	Basidiospores subglobose to broadly ellipsoid, 2.5–3 μm long	***P. cystidiolophora***
24	Basidia > 15 μm long	***P. oreophila***
–	Basidia < 15 μm long	**25**
25	Pores > 10 per mm	***P. fontanesiae***
–	Pores < 10 per mm	**26**
26	Basidia > 5.5 μm long	**27**
–	Basidia < 5.5 μm long	***P. manglietiae***
27	Tramal hyphae interwoven	***P. crataegi***
–	Tramal hyphae subparallel	**28**
28	Pileal surface not radially striate, two black zones present in context	***P. clausenae***
–	Pileal surface radially striate, one black zone present in context	***P. radiata***
29	Cystidioles present	**30**
–	Cystidioles absent	**32**
30	Pores > 9 per mm; basidia > 13 μm long	***P. splendida***
–	Pores < 9 per mm; basidia < 13 μm long	**31**
31	Pores > 7 per mm	***P. osmanthi***
–	Pores < 7 per mm	***P. rigida***
32	Basidia > 19 μm long	***P. cryptolepidis***
–	Basidia < 19 μm long	**33**
33	Basidiospores ellipsoid	**34**
–	Basidiospores cylindrical	***P. cylindrispora***
34	Basidiomata perennial	**35**
–	Basidiomata annual	**36**
35	Pores > 9 per mm	***P. tiliae***
–	Pores < 9 per mm	***P. gutta***
36	Pores > 6 per mm	**37**
–	Pores < 6 per mm	***P. hainaniana***
37	Basidiospores 3–3.5 μm long	***P. minutissima***
–	Basidiospores 3.4–4.1 μm long	***P. weberiana***

## Supplementary Material

XML Treatment for Dentipellicula hainanensis

XML Treatment for Phylloporia rigida

## Additional information

### Use of AI

No use of AI was reported.

### Author ORCIDs

Chang-Ge Song https://orcid.org/0000-0001-5379-2353

### Data availability

All of the data that support the findings of this study are available in the main text.

## References

[B1] Anonymous (1997) The forests in China 1. The Chinese Forest Press, Beijing, 584 pp.

[B2] Bittencourt F, Stürmer SL, Reck MA, Drechsler-Santos ER (2018) *Phylloporia minuta* sp. nov. (Basidiomycota, Hymenochaetales): a remarkable species discovered in a small protected urban area of Atlantic Forest. Phytotaxa 348: 199–210. 10.11646/phytotaxa.348.3.3

[B3] Castro Hernández L, Camino Vilaró M, Herrera Figueroa S (2023) Revisión taxonómica del género *Phylloporia* (Hymenochaetaceae, Basidiomycota) en Cuba. Acta Botánica Mexicana 130: e2149. 10.21829/abm130.2023.2149

[B4] Chamorro-Martínez HA, Raymundo T, Martínez González CR, Acosta EA, Valenzuela R (2022) Two new stipitate species of *Phylloporia* (Basidiomycota, Hymenochaetaceae) from Chamela Biology Station, U.N.A.M. in Jalisco, Mexico. Lilloa 59: 359–375. 10.30550/j.lil/2022.59.S/2022.09.28

[B5] Chen JJ, Shen LL, Dai YC (2015) *Dentipellicula austroafricana* sp. nov. supported by morphological and phylogenetic analyses. Mycotaxon 130: 17–25. 10.5248/130.17

[B6] Chen YY, Zhu L, Xing JH, Cui BK (2017) Three new species of *Phylloporia* (Hymenochaetales) with dimitic hyphal systems from tropical China. Mycologia 109: 951–964. 10.1080/00275514.2017.1410692 29474112

[B7] Cui BK, Yuan HS, Dai YC (2010) Two new species of *Phylloporia* (Basidiomycota, Hymenochaetaceae) from China. Mycotaxon 113: 171–178. 10.5248/113.171

[B8] Cui BK, Li HJ, Ji X, Zhou JL, Song J, Si J, Yang ZL, Dai YC (2019) Species diversity, taxonomy and phylogeny of Polyporaceae (Basidiomycota) in China. Fungal Diversity 97(1): 137–392. 10.1007/s13225-019-00427-4

[B9] Dai YC (2010) Hymenochaetaceae (Basidiomycota) in China. Fungal Diversity 45(1): 131–343. 10.1007/s13225-010-0066-9

[B10] Dai YC (2012) Polypore diversity in China with an annotated checklist of Chinese polypores. Mycoscience 53(1): 49–80. 10.47371/s10267-011-0134-3

[B11] Dai YC, Cui BK, Si J, He SH, Hyde KD, Yuan HS, Liu XY, Zhou LW (2015) Dynamics of the worldwide number of fungi with emphasis on fungal diversity in China. Mycological Progress 14: 62. 10.1007/s11557-015-1084-5

[B12] Decock C, Amalfi M, Robledo G, Castillo G (2013) *Phylloporia nouraguensis*, an undescribed species on Myrtaceae from French Guiana. Cryptogamie. Mycologie 34: 15–27. 10.7872/crym.v34.iss1.2013.15

[B13] Decock C, Yombiyeni P, Memiaghe H (2015) Hymenochaetaceae from the Guineo-Congolian Rainforest: *Phylloporia flabelliforma* sp. nov. and *Phylloporia gabonensis* sp. nov., two undescribed species from Gabon. Cryptogamie. Mycologie 36(4): 449–467. http://www.bioone.org/doi/abs/10.7872/crym/v36.iss4.2015.449

[B14] Dong JH, Li Q, Yuan Q, Luo YX, Zhang XC, Dai YF, Zhou Q, Liu XF, Deng YL, Zhou HM, Muhammad A (2024) Species diversity, taxonomy, molecular systematics and divergence time of wood-inhabiting fungi in Yunnan-Guizhou Plateau, Asia. Mycosphere 15: 1110–1293. 10.5943/mycosphere/15/1/10

[B15] Dong JH, Chen ML, Chen M, Li Q, Zhu Y, Zhang X, Zhou CQ, Li W, Muhammad A, Zhou HM, Jabeen S (2025) Notes, outline, taxonomy and phylogeny of woodinhabiting Agaricales. Mycosphere 16: 2599–2711. 10.5943/mycosphere/16/1/16

[B16] Ferreira-Lopes V, Robledo GL, Reck MA, Góes Neto A, Drechsler Dos Santos ER (2016) *Phylloporia spathulata* sensu stricto and two new South American stipitate species of *Phylloporia* (Hymenochaetaceae). Phytotaxa 257: 133–148. 10.11646/phytotaxa.257.2.3

[B17] Gafforov Y, Tomšovský M, Langer E, Zhou LW (2014) *Phylloporia yuchengii* sp. nov. (Hymenochaetales, Basidiomycota) from Western Tien Shan Mountains of Uzbekistan based on phylogeny and morphology. Cryptogamie. Mycologie 35: 313–322. 10.7872/crym.v35.iss4.2014.313

[B18] Ginns J (1986) The genus *Dentipellis* (Hericiaceae). Windahlia 16: 35–45.

[B19] Hall TA (1999) Bioedit: A user-friendly biological sequence alignment editor and analysis program for windows 95/98/NT. Nucleic Acids Symposium Series 41: 95–98. 10.1021/bk-1999-0734.ch008

[B20] Ipulet P, Ryvarden L (2005) New and interesting polypores from Uganda. Synopsis Fungorum 20: 87–99.

[B21] Jerusalem M, Amalfi M, Yombiyeni P, Castillo G, Decock C (2025) A comprehensive multigene phylogeny of *Phylloporia* (Hymenochaetaceae, Basidiomycota), with an emphasis on tropical African species. Persoonia 54: 1–46. 10.3114/persoonia.2025.54.01 PMC12308284 40746707

[B22] Jerusalem M, Yombiyeni P, Castillo G, Decock C (2019) Hymenochaetaceae (Basidiomycota, Hymenochaetales) from the guineo-congolian phytochorion: Phylloporia rinoreae sp. nov., an additional undescribed species from the forest global earth observatory plot in Gabon. Plant Ecology and Evolution 152: 531–538. 10.5091/plecevo.2019.1567

[B23] Ji XH, Vlasák J, Zhou LW, Wu F, Dai YC (2017) Phylogeny and diversity of *Fomitiporella* (Hymenochaetales, Basidiomycota). Mycologia 109: 308–322. 10.1080/00275514.2017.1305943 28410010

[B24] Katoh K, Standley DM (2013) MAFFT multiple sequence alignment software version 7: Improvements in performance and usability. Molecular Biology and Evolution 30: 772–780. 10.1093/molbev/mst010 PMC3603318 23329690

[B25] Larsson KH, Parmasto E, Fischer M, Langer E, Nakasone KK, Redhead SA (2006) Hymenochaetales: A molecular phylogeny for the hymenochaetoid clade. Mycologia 98: 926–936. 10.1080/15572536.2006.11832622 17486969

[B26] Li M, Ju YH, Jia ZF (2023) *Chaenothecopsis xishuiensis* sp. nov. to science and *Lecanora pseudargentata* newly reported from China. Diversity [14242818] 15(8): 893. 10.3390/d15080893

[B27] Liu JK, Hyde KD, Jones EG, Ariyawansa HA, Bhat DJ, Boonmee S, Maharachchikumbura SS, McKenzie EH, Phookamsak R, Phukhamsakda C, Shenoy BD (2015) Fungal diversity notes 1–100: Taxonomic and phylogenetic contributions to fungal species. Fungal Diversity 72: 1–197. 10.1007/s13225-015-0324-y

[B28] Liu S, Chen YY, Sun YF, He XL, Song CG, Si J, Liu DM, Gates G, Cui BK (2023) Systematic classification and phylogenetic relationships of the brown-rot fungi within the polyporales. Fungal Diversity 118: 1–94. 10.1007/s13225-022-00511-2

[B29] Murrill WA (1904) Shorter Notes. Torrey Botanical Society 4: 141–143.

[B30] Nylander JAA (2004) MrModeltest v2. Program. Distributed by the Author; Evolutionary Biology Center. Uppsala, Sweden.

[B31] Olou BA, Ordynets A, Langer E (2019) First new species of *Fulvifomes* (Hymenochaetales, Basidiomycota) from tropical Africa. Mycological Progress 18: 1383–1393. 10.1007/s11557-019-01536-9

[B32] Olou BA, Yorou NS, Langer E (2021) New species and a new record of *Phylloporia* from Benin. Scientific Reports 11: 1–15. 10.1038/s41598-021-88323-3 PMC8065173 33893360

[B33] Olou BA, Krah FS, Piepenbring M, Yorou NS (2023) *Phylloporia mutabilis* sp. nov. from Benin, West Africa. Fungal Systematics and Evolution 12: 81–89. 10.3114/fuse.2023.12.06 PMC10964571 38533479

[B34] Posada D, Crandall KA (1998) Modeltest: Testing the model of DNA substitution. Bioinformatics 14: 817–818. 10.1093/bioinformatics/14.9.817 9918953

[B35] Qin WM, Wang XW, Sawahata T, Zhou LW (2018) *Phylloporia lonicerae* (Hymenochaetales, Basidiomycota), a new species on *Lonicera japonica* from Japan and an identification key to worldwide species of *Phylloporia*. MycoKeys 30: 17–30. 10.3897/mycokeys.30.23235 PMC5904531 29681730

[B36] Rajchenberg M, Pildain MB, Madriaga DC, de Errasti A, Riquelme C, Becerra J (2019) New poroid Hymenochaetaceae (Basidiomycota, Hymenochaetales) from Chile. Mycological Progress 18: 865–877. 10.1007/s11557-019-01495-1

[B37] Rathnayaka AR, Tennakoon DS, Jones GE, Wanasinghe DN, Bhat DJ, Priyashantha AH, Stephenson SL, Tibpromma S, Karunarathna SC (2025) Significance of precise documentation of hosts and geospatial data of fungal collections, with an emphasis on plant-associated fungi. New Zealand Journal of Botany 63: 462–489. 10.1080/0028825X.2024.2381734

[B38] Ren GJ, Wu F (2017) *Phylloporia lespedezae* sp. nov. (Hymenochaetaceae, Basidiomycota) from China. Phytotaxa 299: 243–251. 10.11646/phytotaxa.299.2.8

[B39] Ronquist F, Huelsenbeck JP (2003) MrBayes 3: Bayesian phylogenetic inference under mixed models. Bioinformatics 19: 1572–1574. 10.1093/bioinformatics/btg180 12912839

[B40] Sayers EW, Cavanaugh M, Clark K, Pruitt KD, Schoch CL, Sherry ST, Karsch-Mizrachi I (2020) GenBank. Nucleic Acids Research 48: D84–D86. 10.1093/nar/gkz956 PMC7145611 31665464

[B41] Shao YY, Zheng H, Huang HS, Wei QL, Huang FC, Liu B (2025) Two new species of *Phylloporia* (Hymenochaetaceae, Basidiomycota) from the karst region in southern China. Phytotaxa 693(3): 221–234. 10.11646/phytotaxa.693.3.3

[B42] Song CG, Sun YF, Wu DM, Gao N, Liu S, Xu TM, Cui BK (2022) Morphology and molecular phylogeny reveal five new species of *Hydnellum* (Bankeraceae, Thelephorales) from China. Frontiers in Microbiology 13: 1049007. 10.3389/fmicb.2022.1049007 PMC9683478 36439794

[B43] Song CG, Sun YF, Liu S, Chen YY, Cui BK (2023) Phylogenetic analyses and morphological studies reveal four new species of *Phellodon* (Bankeraceae, Thelephorales) from China. Journal of Fungi 9: 30. 10.3390/jof9010030 PMC9861862 36675852

[B44] Song CG, Xu TM, Xu YH, Wang D, Zeng L, Fan XP, Sun YF, Cui BK (2025) Systematic revision, molecular phylogeny and divergence times of Thelephorales (Basidiomycota). Mycosphere 16(1): 296–422. 10.5943/mycosphere/16/1/5

[B45] Stamatakis A (2006) RAxML-VI-HPC: Maximum likelihood-based phylogenetic analysis with thousands of taxa and mixed models. Bioinformatics 22: 2688–2690. 10.1093/bioinformatics/btl446 16928733

[B46] Valenzuela R, Raymundo T, Cifuentes J, Castillo G, Amalfi M, Decock C (2011) Two undescribed species of *Phylloporia* from Mexico based on morphological and phylogenetic evidence. Mycological Progress 10: 341–349. 10.1007/s11557-010-0707-0

[B47] Wagner T, Fischer M (2002) Proceedings towards a natural classification of the worldwide taxa *Phellinus* s.l. and *Inonotus* s.l., and phylogenetic relationships of allied genera. Mycologia 94: 998–1016. 10.1080/15572536.2003.11833156 21156572

[B48] Wagner T, Ryvarden L (2002) Phylogeny and taxonomy of the genus *Phylloporia* (Hymenochaetales). Mycological Progress 1: 105 116. 10.1007/s11557-006-0009-8

[B49] Wang M (2018) Taxonomy and phylogeny of dentate fungi in Hericiaceae from China. PhD Thesis, University of Beijing Forestry University, Beijing, China.

[B50] Wu F, Ren GJ, Wang L, Oliveira-Filho JRC, Gibertoni TB, Dai YC (2019a) An updated phylogeny and diversity of *Phylloporia* (Hymenochaetales): Eight new species and keys to species of the genus. Mycological Progress 18(5): 615–639. 10.1007/s11557-019-01476-4

[B51] Wu F, Dai SJ, Vlasák J, Spirin V, Dai YC (2019b) Phylogeny and global diversity of *Porodaedalea*, a genus of gymnosperm pathogens in the Hymenochaetales. Mycologia 111: 40–53. 10.1080/00275514.2018.1526618 30640586

[B52] Wu SH, Chang CC, Wei CL, Lin YT, Chen SZ (2020) Four new species of *Phylloporia* (Hymenochaetales, Basidiomycota) from Southeastern Taiwan. Mycological Progress 19: 743–752. 10.1007/s11557-020-01590-8

[B53] Wu SH, Chang CC, Wei CL, Jiang GZ (2021) *Phylloporia moricola* sp. nov. (Hymenochaetales, Basidiomycota) from China. Phytotaxa 501: 181–188. 10.11646/phytotaxa.501.1.9

[B54] Wu F, Man XW, Tohtirjap A, Dai YC (2022a) A comparison of polypore funga and species composition in forest ecosystems of China, North America, and Europe. Forest Ecosystems 9: 100051. 10.1016/j.fecs.2022.100051

[B55] Wu F, Zhou LW, Vlasák J, Dai YC (2022b) Global diversity and systematics of Hymenochaetaceae with poroid hymenophore. Fungal Diversity 113: 1–192. 10.1007/s13225-021-00496-4

[B56] Yang Y, Xu Y, Wang L, Jiang QQ, Su JQ, Li R, Zhou HM, Zhao CL (2025) Multigene phylogeny of seven wood-inhabiting fungal orders in Basidiomycota, and proposal of a new genus and thirteen new species. Mycosphere 16: 245–295. 10.5943/mycosphere/16/1/4

[B57] Yombiyeni P, Decock C (2017) Hymenochaetaceae (Hymenochaetales) from the Guineo-Congolian phytochorion: *Phylloporia littoralis* sp. nov. from coastal vegetation in Gabon, with an identification key to the local species. Plant Ecology and Evolution 150: 160–172. 10.5091/plecevo.2017.1289

[B58] Yombiyeni P, Balezi A, Amalfi M, Decock C (2015) Hymenochaeta ceae from the Guineo-Congolian rainforest: Three new species of *Phylloporia* based on morphological, DNA sequences and ecological data. Mycologia 107: 996–1011. 10.3852/14-298 26240304

[B59] Yu HY, Zhao CL, Dai YC (2013) *Inonotus niveomarginatus* and *I. tenuissimus* spp. nov. (Hymenochaetales), resupinate species from tropical China. Mycotaxon 124: 61–68. 10.5248/124.61

[B60] Yuan Y, Bian LS, Wu YD, Chen JJ, Wu F, Liu HG, Zeng GY, Dai YC (2023) Species diversity of pathogenic wood-rotting fungi (Agaricomycetes, Basidiomycota) in China. Mycology 14: 204–226. 10.1080/21501203.2023.2238779 PMC10424591 37583455

[B61] Zhou LW (2013) *Phylloporia tiliae* sp. nov. from China. Mycotaxon 124: 361–365. 10.5248/124

[B62] Zhou LW (2014) Notes on the taxonomic positions of some Hymenochaetaceae (Basidiomycota) species with colored basidiospores. Phytotaxa 177: 183. 10.11646/phytotaxa.177.3.7

[B63] Zhou LW (2015a) Four new species of *Phylloporia* (Hymenochaetales, Basidiomycota) from tropical China with a key to *Phylloporia* species worldwide. Mycologia 107: 1184–1192. 10.3852/14-254 26297774

[B64] Zhou LW (2015b) *Phylloporia osmanthi* and *P. terrestris* spp. nov. (Hymenochaetales, Basidiomycota) from Guangxi, South China. Nova Hedwigia 100: 239–249. 10.1127/nova_hedwigia/2014/0220

[B65] Zhou LW (2016) *Phylloporia minutipora* and *P. radiata* spp. nov. (Hymenochaetales, Basidiomycota) from China and a key to worldwide species of *Phylloporia*. Mycological Progress 15: 1–11. 10.1007/s11557-016-1200-1

[B66] Zhou LW, Dai YC (2012) Phylogeny and taxonomy of *Phylloporia* (Hymenochaetales): New species and a worldwide key to the genus. Mycologia 104: 211–222. 10.3852/11-093 21933921

[B67] Zhou LW, Dai YC (2013) Taxonomy and phylogeny of hydnoid Russulales: Two new genera, three new species and two new combination species. Mycologia 105: 636–649. 10.3852/12-011 23360974

[B68] Zhou M, Chen JJ, Vlask J, Yuan Y (2021) *Dentipellicula guyanensis* sp. nov. (Hericiaceae, Basidiomycota) from French Guiana. Phytotaxa 478(2): 261–267. 10.11646/phytotaxa.478.2.7

[B69] Zhou M, Wu F, Dai YC, Vlasák J (2022) Two new species of *Phylloporia* (Hymenochaetales) from the Neotropics. MycoKeys 90: 71–83. 10.3897/mycokeys.90.84767 PMC9849063 36760419

